# Effects of pendulum appliance versus clear aligners in the vertical dimension during Class II malocclusion treatment: a randomized prospective clinical trial

**DOI:** 10.1186/s12903-022-02483-w

**Published:** 2022-10-10

**Authors:** Roberta Lione, Alessia Balboni, Valentina Di Fazio, Chiara Pavoni, Paola Cozza

**Affiliations:** 1 Department of Systems Medicine, University of Rome ‘Tor Vergata’, Viale Oxford, 81, 00133 Rome, Italy; 2 Department of Dentistry, UNSBC, Tirana, Albania; 3 Department of Faculty of Medicine and Surgery, UniCamillus International Medical University, Rome, Italy

**Keywords:** Class II, Distalization, Pendulum, Clear aligners

## Abstract

**Background:**

The aim of the present study was to compare the effects on vertical dentoskeletal dimension produced by Pendulum appliance and Clear Aligners in patients with Class II malocclusion.

**Trial design:**

This is a prospective two-arm parallel group randomized clinical trial with 1:1 allocation ratio.

**Methods:**

The Pendulum Group (PG) consisted of 20 patients (15F, 5 M) with a mean age of 17.2 ± 4.3 years. The Clear Aligners Group (CAG) comprised 20 patients (13F, 7 M) with a mean age of 17.2 ± 3.2 years. Distalization’s protocol in PG involved the activation of TMA wires till the achievement of Class I molar relationship. A protocol of sequential distalization was applied in the CAG. For each subject lateral cephalograms have been analyzed before treatment (T1) and at the end of the therapy (T2). Descriptive statistics and statistical between-group comparisons (PG vs CAG) were calculated for the craniofacial starting forms at T1 and for the T2–T1 changes. Statistical between-group comparisons for the T2–T1 changes were performed with independent samples *t*-tests (*P* < 0.05).

**Results:**

The PG showed significantly greater increases in SN^GoGn° when compared with CAG (+ 2.1 and − 0.3 degrees, respectively). Clockwise rotation of the occlusal plane with significantly greater increase of SN^POccl angle was observed in PG (+ 2.8 degrees) when compared with CAG (− 4.2 degrees).

The PG revealed a significant increase in the N-Me variable with a mean change of + 4.4 mm compared to the CAG with mean values of − 1.2 mm. The PG showed an increase in the ArGo^GoMe angle (+ 0.7° degrees) compared to the CAG (− 3.4° degrees). The PG showed significantly greater increases in both maxillary and mandibular first molar to palatal plane (+ 1.3 and + 2.1 mm, respectively) when compared with CAG (− 0.9 and − 0.2 mm, respectively).

**Conclusions:**

Upper molar distalization with clear aligners represents a valid alternative to non-extraction treatment of Class II malocclusion, reducing the extrusion of maxillary first molars and improving the management of the occlusal plane and vertical dimension.

*Trial registration*: ClinicalTrials.gov, NCT05298280. Registered 28 March 2022—Retrospectively registered, https://clinicaltrials.gov.

## Background

Treatment of Class II malocclusion is one of the most investigated and controversial issues in contemporary orthodontics because of the extensive variability of treatment protocols addressing the morphological characteristics of it [1, 2]. Maxillary molar distalization is one of the most common strategies to correct Class II molar relationship and it is commonly indicated for patients with maxillary dentoalveolar protrusion or minor skeletal discrepancies [3, 4]. In 1992 Hilgers introduced the Pendulum appliance that has shown great results in terms of molars distalization. However, many side effects such as labial/mesial tipping and protrusion of the maxillary incisors and premolars, distal tipping of the maxillary molars, increase in lower anterior face height, clockwise mandibular rotation and extrusion of the first premolars have been reported [5–11]. During the following phase with fixed appliance the side-effects have to be corrected [5–9].

In the last decades, the orthodontic treatment with removable clear aligners has become an increasing common choice because of the growing number of adult patients who ask for aesthetic and comfortable alternatives to conventional fixed appliances [12–14].

Only few investigations have focused on the predictability of orthodontic tooth movement with clear aligners (CAT) [15–17]. A systematic review by Rossini et al. pointed out that among the dental movements analyzed in 11 studies, the bodily distalization was the most predictable [17].

According to Simon et al. aligners allow a high accuracy (88%) of the bodily movement of upper molars when a mean distalization movement of 2.7 mm was required, especially when the movement was assisted by the use of attachments [18, 19].

As reported by Ravera et al. maxillary distalization without mesiodistal tipping movements and lower facial height changes can be achieved with clear aligners. Consequently, the use of aligners is recommended when 2 to 3 mm of maxillary molar distalization are needed in non-growing subjects [14].

However, a detailed analysis of the skeletal and dental changes produced by pendulum appliance and by clear aligners in Class II treatment is still lacking. The hypothesis underlying this investigation is that the presence of plastic coverage on posterior upper and lower teeth provided by clear aligners allows for a better control of side effects on vertical dimension.

Therefore, the aim of the present randomized prospective clinical study was to compare the effects on vertical dimension determined by maxillary molar distalization with pendulum versus clear aligners at the end of comprehensive treatment.

## Methods

### Study design

The Consolidated Standards of Reporting Trials (CONSORT) checklist was used as a guideline for conducting and reporting this trial. The present RCT was designed as a prospective two-arm parallel group randomized clinical trial with 1:1 allocation ratio. The study was carried out in accordance with the Declaration of Helsinki and the proposal was approved by the Ethics Committee at the University of XXXX (protocol number 257/21). After a full explanation of the nature, purpose, and material risks of the proposed procedures, informed consent was obtained from patients or from patients’ parents for juvenile subjects. The patients were aware that they could have received a different treatment method according to a randomized allocation sequence. The trial was registered on ClinicalTrials.gov (registration number: NCT05298280).

### Population

All subjects were selected according to the following inclusion criteria: permanent dentition with upper and lower second molars fully erupted, bilateral Class II or end to end Class II molar relationship (up to 3 mm of molar relationship discrepancy), skeletal Class I or mild Class II malocclusion (ANB angle between 2 and 7°), normodivergence on the vertical plane (SN^GoGn comprised between 32 and 37 degrees), crowding in the lower arch (≤ 6 mm). All patients were in good general health with healthy periodontium, generalized probing depths not exceeding 3 mm, and no radiographic evidence of periodontal bone loss. The exclusion criteria were: patients who required functional appliance therapy, those who had previous orthodontic treatment or extraction including extraction of upper third molars, hypodontia, craniofacial syndromes or cleft, previous prosthodontic treatments of the upper molars.

### Treatment

Subjects enrolled in the study were randomly assigned to two groups: Pendulum Group (PG) and Clear Aligner Group (CAG). All subjects were treated by the same clinician (RL).

### Pendulum group (PG)

**Fig. 1:**
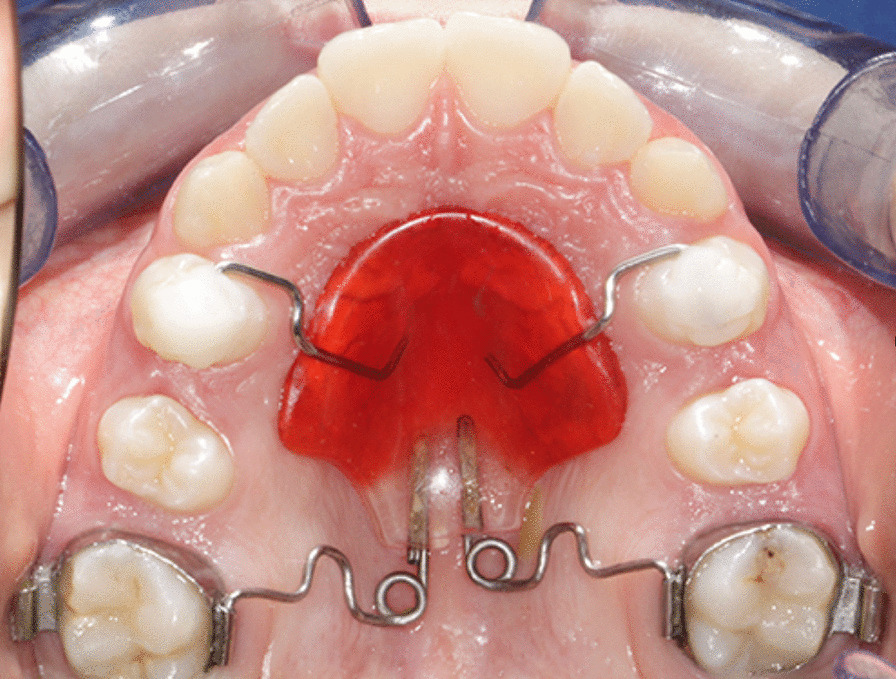
Treatment protocol with Pendulum appliance

In the PG, all patients received a pendulum appliance as described by Angelieri et al. [20]. The Nance button was anchored to the first and second premolars with removable wires. The 0.032-inch TMA wires were activated 45 degrees to produce a force of 200–250 g per side. On average, intraoral reactivation of the distalizing springs was performed twice during the procedure. As recommended by Byloff et al*.*, an uprighting bend was added to the end of TMA wire to prevent excessive molar tipping. When a super Class I molar relationship was obtained, pendulum was replaced by a Nance holding arch. The average treatment duration was 6 months followed by full fixed conventional 0.022-in slot pre-adjusted edgewise brackets (McLaughlin–Bennett–Trevisi prescription) on all teeth including upper and lower second molars. Standard continuous archwire sequence during alignment and levelling phases (0.016-in round, 0.019 × 0.025-in rectangular, nickel–titanium alloys and 0.019 × 0.025 in stainless steel) was used in all subjects. Alignment and levelling were considered finished when passive engagement of a 0.019 × 0.025-in stainless steel archwire was obtained. All subjects were treated with Class II intermaxillary elastics (1/4 in., 6.5 oz) after the achievement of Class I molar relationship to support anchorage in addition to Nance button during the retraction of upper premolars, canines and incisors. The patients were instructed to wear Class II elastics for at least 16 h per day. The Class II elastics were dismissed at the end of the working phase. The mean treatment time was 20 ± 2 months (Fig. 1).

### Clear aligner group (CAG)

The treatment of sequential upper arch distalization was performed by the same board-certified orthodontists as proposed by Align Technology and described by Ravera et al*.* [14]*.*

**Fig. 2:**
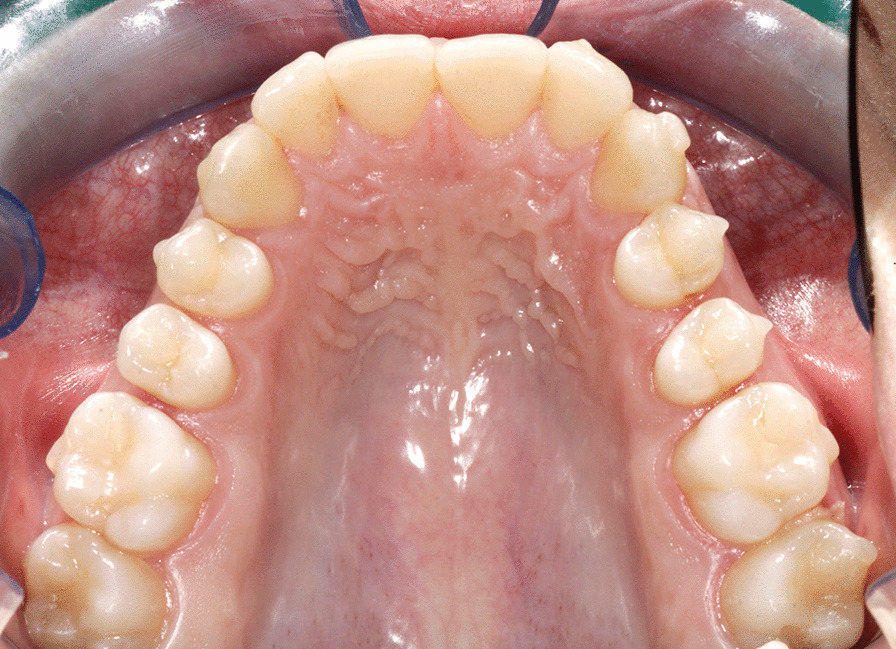
Treatment protocol with Clear aligners

The standardized orthodontic intervention was represented by the maxillary molar distalization protocol proposed by Align Technology: the ClinCheck® of each treated case was planned and approved by a single trained orthodontist (RL) in order to obtain a sequential distalization on the upper arch and the staging was set at 0.25 mm per aligner. The distalization starts with the upper second molars; when the second molars are halfway, the upper first molars move back, then premolars, and so on until the “en masse” retraction of the four incisors completes the treatment plan [21]. The protocol involves the use of attachments and Class II elastics. Intermaxillary elastics were used during the retraction of premolars, canines, and incisors. In order to control the distalization movements, the same rectangular not beveled and vertical attachments with a length of 3 mm were placed on the distalizing teeth of all patients (from canine to second molar) [22, 23]. In a sequential distalization setup, distalization is built into the aligners and it is the aligner that moves the teeth back, not the elastics [21]. As the molars are distalized with the aligners, they are pitted against the rest of the arch for anchorage. To prevent loss of anchorage and thus possible flaring of the anterior teeth, Class II elastics (1/4 in., 6.5 oz) from buttons on upper canines to precision cuts at the level of lower first molars are used to reinforce the anchorage during the retraction of upper premolars, canines and incisors. Patients selected for the study satisfied the compliance criteria of wearing aligners and Class II elastics at least 20–22 h per day with regular 4-week monitoring. At each visit, the patient was motivated to obtain a proper wearing of both elastics and aligners. As well as for the PG, the Class II elastics were dismissed at the end of the working phase. Interproximal reduction was not applied. The average number of required aligners was 57 ± 5 on the upper and lower arch. Each couple of aligners was worn for 7 days. At the end of distalization all patients needed a refinement phase, corresponding to the finishing phase, that was performed with a mean number of 19 ± 5 aligners. During the refinement phase, each aligner was worn for 7 days. The mean treatment time was in total 18 ± 4 months (Fig. 2).

### Outcome measurements

**Fig. 3:**
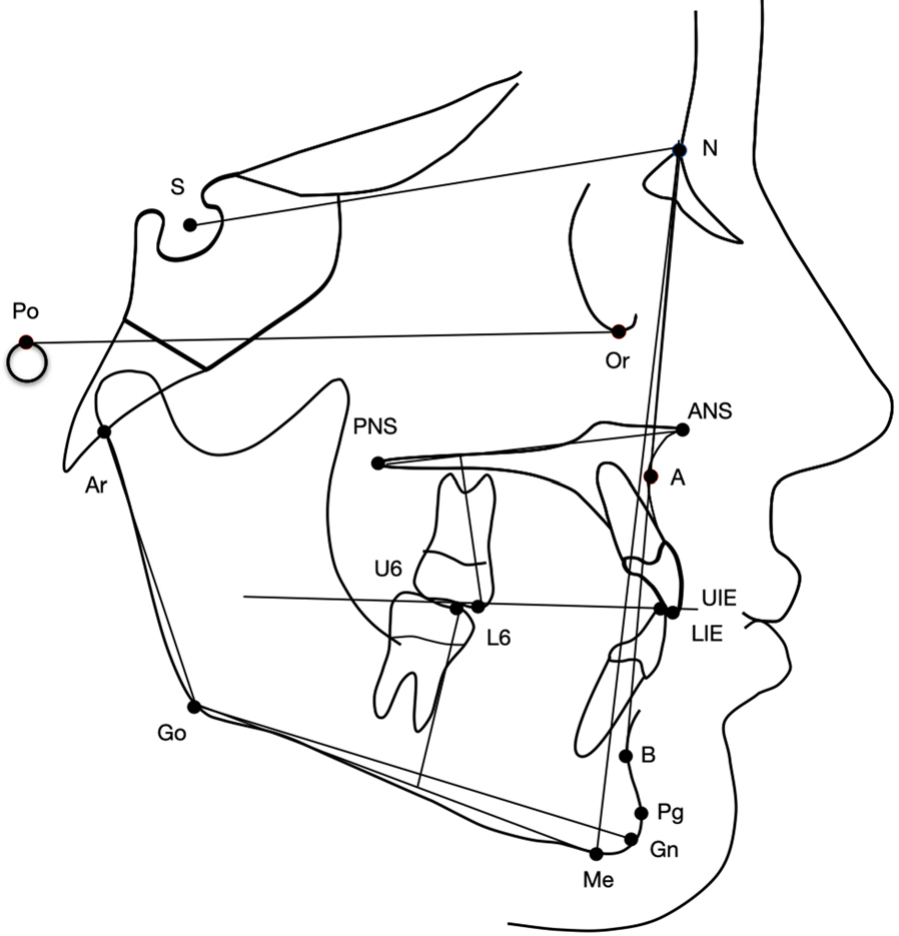
Cephalometric points, lines, and angles used in analysis: SNA angle; SNB angle; ANB angle; Ar-Go to mandibular plane (Go-Me) angle; upper anterior facial height (N-ANS); lower anterior facial height (ANS-Me); anterior facial height (N-Me); maxillary first molar (6/) to palatal plane (ANS-PNS); mandibular first molar (/6) to mandibular plane (Me-Go); overbite; overjet

For both treatment groups, lateral cephalograms were required before treatment (T1) and at the end of the comprehensive therapy (T2) with a mean interval of 2.1 years between the two observation times. All lateral cephalograms were hand traced at a single sitting. Cephalograms were traced and landmark identification was performed by one investigator. A customized digitization template (Viewbox, version 3.0, dHAL Software, Kifissia, Greece) was created and used for the cephalometric evaluation. For each patient lateral cephalograms at T1 and T2 were digitized, and a custom cephalometric analysis was used. Fourteen variables (7 linear and 7 angular) were generated for each tracing [24]. Standard cephalometric landmarks were then identified on each radiograph (Fig. 3).

### Randomization, allocation concealment and blinding

A computer-generated random number list was used to allocate patients to treatments. Block randomization was used to assign the same number of patients to each treatment. The allocation sequence was concealed by the statistician, who used opaque and sealed envelopes, sequentially numbered for each patient. The observer (AB) who performed all the measurements was blinded to the group assignment. The study was blinded in regard to the statistical analysis: blinding was obtained by eliminating from the elaboration file every reference to patient group assignment.

### Sample size

A sample size for this trial was calculated according to the method proposed by Whitehead et al. [25]. For an effect size of 1 for the primary outcome variable SN^GoGn°, a sample size of 17 subjects per group was required for a type I error rate of 5% and a power of 80%. To account for potential dropouts, 20 subjects per group were recruited.

### Statistical analysis

To determine the reliability of the method, 15 randomly selected radiographs were traced and digitized by the same investigator on two separate occasions at least 1 month apart. A paired *t*-test was used to compare the two measurements (systematic error). The magnitude of the random error was calculated by using the method of moment’s estimator (MME) [26].

The primary outcome was considered the changes in total vertical dimension (SN^GoGn°) while secondary outcome was considered reduced Overjet. Exploratory statistics revealed that all cephalometric variables were normally distributed (Kolmogorov–Smirnov test) with equality of variances (Levene’s test).

Descriptive statistics and statistical between-group comparisons (PG vs CAG) were calculated for the craniofacial starting forms at T1 and for the T2–T1 changes. Statistical between-group comparisons for the T2–T1 changes were performed with independent samples *t*-tests. The significance level was set at *P* < 0.05. All statistical computations were performed with SPSS software (Statistical Package for the Social Sciences, SPSS, version 12, Chicago, Illinois, USA).

## Results

No systematic error was found between the repeated cephalometric values. For the cephalometric variables, the random error varied from 0.24 (SNA angle) to 0.37° (gonial angle) for angular measurements and from 0.17 (overjet) to 0.24 mm (overbite) for linear measurements.

**Fig. 4:**
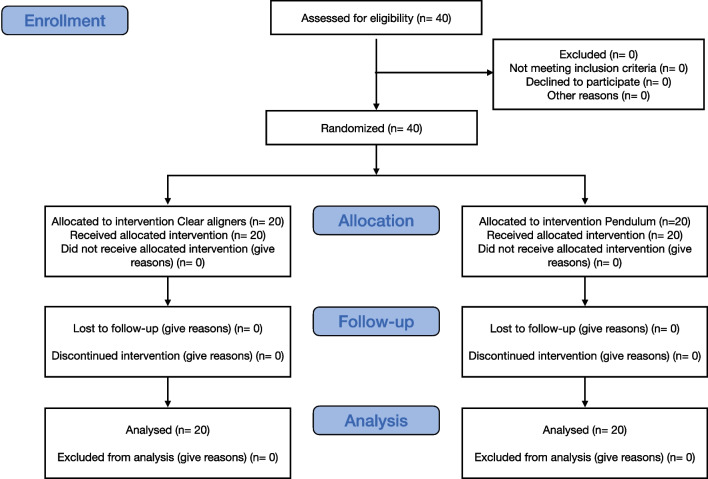
CONSORT flow diagram

40 patients were randomly assigned to the interventions, 20 patients were treated with pendulum appliance and the other 20 were treated with Invisalign orthodontic aligners (Invisalign, Align Technology, San Josè, California, USA). The recruitment started in January 2020 and the observation period ended in June 2022 (Fig. 4).

PG included 20 patients (15 females and 5 males) with a mean age of 17.2 ± 4.3 years; 7 patients with bilateral Class II (2.7 ± 0.5 mm) and 13 patients with bilateral end to end Class II molar relationship (2.3 ± 0.5 mm). The CAG comprised 20 subjects (13 females, 7 males) treated with clear aligners with a mean age of 17.1 ± 3.2 years; 6 patients with bilateral Class II (2.7 ± 0.5 mm) and 14 patients with bilateral end to end Class II molar relationship (2.3 ± 0.5 mm).

As reported in Table 1, the analysis of the starting forms showed no statistically significant differences at T1 between the groups for all the performed measurements.

**Table 1:** Descriptive statistics and statistical comparisons (independent-samples *t*-tests) of the starting forms (cephalometric values at T1)

Variables	Pendulum group T1		Clear aligner group T1				95% CI of the difference	
	n=20	f=15; m=5	n=20	f=13; m=7				
	Mean	SD	Mean	SD	Difference	*P*.Value	Lower	Upper
Age y	17.2	4.3	17.1	3.2	0.1	NS	− 2.3	2.5
SNA°	82.4	4	81.8	3.9	0.6	NS	− 2.2	3.4
SNB°	77.8	3.8	76.8	3.7	1	NS	− 1.7	3.7
ANB°	4.6	2.3	5.1	1.8	− 0.5	NS	− 2.0	1.0
SN^GoGn°	31.5	6	29.6	5.3	1.9	NS	− 2.3	6.1
ArGo^GoMe	126.6	6	123.8	5.3	2.2	NS	− 1.4	7.0
N-Me mm	106.5	8.5	111.9	5	− 5.9	NS	− 10.9	0.7
N-ANS mm	46.3	4.1	48.1	5.2	− 1.8	NS	− 5.1	1.5
ANS-Me mm	60.2	5.3	63.9	5.4	− 3.7	NS	− 7.5	0.1
SN^POccl°	18.4	5.3	17.4	6.8	1	NS	− 3.2	5.2
SN^ANS-PNS°	6.7	2.5	8.7	3.2	2	NS	− 4.0	0.0
OVJ mm	5.3	2.1	4.7	1.5	0.6	NS	− 0.7	1.9
OVB mm	2.2	1.9	2.7	1.9	0.5	NS	− 1.8	0.8
L6-GoMe mm	28.5	2.5	30.3	2.4	− 1.8	NS	− 3.6	0.0
U6-ANSPNS mm	20.7	1.8	21	1.1	− 1.3	NS	− 2.2	0.0
Sum mm	49.2	1.1	51.3	1.4	− 3.1	NS		

Sum indicates maxillary first molar to palatal plane + mandibular first molar to mandibular plane

*NS* not significant, *CI* confidence of interval, *SD* standard deviation

*P < 0.5, **P < 0.01, ***P < 0.001

Both distalizing protocols resulted effective in achieving a Class I molar relationship and the correction of overjet at the end of the active therapy (T2).

Descriptive statistics and statistical comparisons on the T2–T1 changes are given in Table 2. The PG showed significantly greater increases after distalization (*P* < 0.01) in SN^GoGn° when compared with CAG (+ 2.1 and − 0.3 degrees, respectively). Clockwise rotation of the occlusal plane with significantly greater increase of SN^POccl angle was observed (*P* < 0.001) in PG (+ 2.8 degrees) when compared with CAG (− 4.2 degrees).

**Table 2:** Descriptive statistics and statistical comparisons (independent-samples *t*-tests) of the T2–T1 changes

Variables	Pendulum group		Clear aligner group				95% CI of the difference	
	n = 20	f = 15; m = 5	n = 20	f = 13; m = 7				
	Mean	SD	Mean	SD	Difference	*P. *value	Lower	Upper
SNA°	0.4	2.2	0.2	1.9	0.2	NS	− 0.7	2.3
SNB°	0.1	2.1	1.1	1.1	− 1	NS	− 0.1	2.7
ANB°	0.8	1.4	1.2	1.2	− 0.4	NS	− 1.4	0.6
SN^GoGn°	2.1	1.8	− 0.3	1.5	2.4	**	− 2.4	− 0.1
ArGo^GoMe°	0.7	4.6	− 3.4	2.9	4.1	*	− 5.6	− 0.2
N-Me mm	4.4	6.1	− 1.2	3.1	5.6	*	− 6.2	− 0.7
N-ANS mm	0.8	5.3	− 1.2	2.1	2	NS	− 5.1	1.2
ANS-Me mm	3.0	4.4	− 0.6	3.0	3.6	NS	− 5.3	0.4
SN^POccl °	2.8	2.9	− 4.2	3.0	7.0	***	− 9.3	− 4.7
SN^ANS-PNS °	0.3	2.5	− 1.1	1.9	1.4	NS	− 3.2	0.2
OVJ mm	− 1.2	2.5	− 1.3	0.9	0.1	NS	0.85	2.2
OVB mm	1.2	1.8	1.2	1.2	0	NS	− 1.1	1.2
L6-GoMe mm	2.1	2.4	− 0.2	1.2	2.3	**	− 3.4	− 0.4
U6-ANSPNS mm	1.3	3.0	− 0.9	0.8	2.2	**	− 3.9	− 0.4

*NS* not significant, *CI* confidence of interval

* < 0.05, ** < 0.01, *** < 0.001

Finally, differences were observed in anterior facial height (N-Me) (*P* < 0.05) and in the ArGo^GoMe angle (*P* < 0.05). The PG revealed a significant increase in the N-Me variable with a mean change of + 4.4 mm compared to the CAG with mean values of − 1.2 mm. The PG showed an increase in the ArGo^GoMe angle (+ 0.7° degrees) compared to the CAG (− 3.4° degrees). The PG showed significantly greater increases (*P* < 0.01) in both maxillary and mandibular first molar to palatal plane and to mandibular plane (+ 1.3 and + 2.1 mm, respectively) when compared with CAG (− 0.9 and − 0.2 mm, respectively).

No significant between-group differences were recorded for any of the other sagittal skeletal variables or for the other dentoalveolar variables.

## Discussion

The two treated groups were matched according to gender, age, observation times, and occlusal characteristics and no statistically significant differences resulted in all cephalometric variables investigated (Table 1).

To our knowledge, no previous papers in literature have compared the vertical treatment changes of both devices.

Several studies observed that pendulum followed by conventional fixed appliances caused undesired effects during upper molars distalization such as molar distal tipping, molar extrusion, clockwise rotation of the mandibular plane, and increase in the anterior facial height [11, 20, 27–30].

On the contrary, only few studies investigated the predictability and the effects of molar distalization with CAT. Ravera et al. analyzed a group of 32 Caucasian subjects treated with Invisalign, highlighting that the distalization movement was not associated with significant distal tipping of the distalized molars due to rectangular and vertical attachments necessary to create a sufficient moment to oppose the tipping movements. In the same way, the distal molar movement was not associated with extrusion of the teeth [14].

Caruso et al. analyzed the sagittal and vertical dimension changes in a group of 10 subjects treated by sequential upper molar distalization performed with CAT. The authors reported that no changes in total divergence were observed during distal bodily movement of upper molars [31].

In addition, no significant rotation of the occlusal plane was recorded.

In the present study, both distalizing protocol resulted effective in achieving a Class I molar relationship and the correction of overjet at the end of active therapy (T2).

In agreement with the literature a slight extrusion of the maxillary molars (+ 1.3 mm) and mandibular molars (+ 2.1 mm), clockwise rotation of the occlusal plane (+ 2.8 degrees), and an increase in anterior facial height (+ 4.4 mm) were observed in PG [6, 7, 10, 32]. The PG showed significantly greater increases at T2 (*P* < 0.01) in SN^GoGn° when compared with CAG (+ 2.4 mm). The present findings suggested that clear aligners allowed a good control of divergence during molar distalization. Skeletal vertical dimension was not affected by the distalization of maxillary molars with aligners and these results are in according to what reported by Ravera in 2016 and Caruso in 2019 [14, 31].

Moreover, results from the current study showed clockwise rotation of the occlusal plane with significantly greater increase of SN^POccl° (+ 7.0 degrees) in PG when compared with CAG (*P* < 0.001). These findings indicate better occlusal plane control in patients treated with CAT, probably due to less molar tipping achieved during distalization and due to less upper and lower molar extrusion (U6-ANSPNS + 2.2 mm; L6-GoMe + 2.3 mm; *P* < 0.01).

Finally, differences were observed in anterior facial height (N-Me) (*P* < 0.05) and in the ArGo^GoMe angle (*P* < 0.05). The PG revealed a significant increase in the N-Me variable (+ 5.6 mm) and in gonial angle (+ 4.1 degrees) when compared to CAG.

Vertical growth pattern is an important factor to consider in Class II treatment with molar distalization. Molar extrusion and clockwise rotation of the occlusal plane, in fact, can lead to a worsening of the profile and cause open bite. According to our results, distalization treatment with CAT represents an effective alternative to Class II treatment and it is associated with better control of occlusal plane rotation and vertical dimension when compared with pendulum appliance. The thickness of the aligner and the resulting “bite block” effect could explain the absence of a significant increase in vertical dimension.

## Limitations

A limitation of this study was a relatively short-term follow-up. Indeed, a long-term observation is required to support our claim that Clear Aligners improves vertical dimension management. Moreover, although intrusion movements were not planned or performed during the retraction of lateral and anterior upper segments, the effects of distalization with the two different treatment protocols, were assessed at the end of comprehensive treatment. Further studies are necessary to detect the changes of vertical dimension at the end of distalization phase.

## Generalizability

The results of the present study can be generalized for patient groups with similar mean age, inclusion/exclusion criteria, and treatment protocol.

## Harms

No harms or other important unintended consequences were observed during the trial.

## Conclusions

CAT is effective in distalizing maxillary molars with a better control of vertical dimension, occlusal plane rotation, and molar extrusion when compared with pendulum appliance.

## Data Availability

The datasets used and/or analysed during the current study are available from the corresponding author on reasonable request.

## Abbreviations

PG: Pendulum group
CAG: Clear aligners group
CAT: Clear aligners treatment
